# The Interactions of Human Neutrophils with Shiga Toxins and Related Plant Toxins: Danger or Safety?

**DOI:** 10.3390/toxins4030157

**Published:** 2012-03-01

**Authors:** Maurizio Brigotti

**Affiliations:** Dipartimento di Patologia Sperimentale, Università di Bologna, Via San Giacomo 14, 40126 Bologna, Italy; Email: maurizio.brigotti@unibo.it; Tel.: +39-051-2094716; Fax: +39-051-2094746

**Keywords:** Shiga toxins, ricin, ribosome-inactivating proteins, polymorphonuclear leukocytes, hemolytic uremic syndrome

## Abstract

Shiga toxins and ricin are well characterized similar toxins belonging to quite different biological kingdoms. Plant and bacteria have evolved the ability to produce these powerful toxins in parallel, while humans have evolved a defense system that recognizes molecular patterns common to foreign molecules through specific receptors expressed on the surface of the main actors of innate immunity, namely monocytes and neutrophils. The interactions between these toxins and neutrophils have been widely described and have stimulated intense debate. This paper is aimed at reviewing the topic, focusing particularly on implications for the pathogenesis and diagnosis of hemolytic uremic syndrome.

## 1. Shiga Toxins and Ricin Are Analogous Toxins

It is intriguing that two quite different biological kingdoms have evolved, in parallel, the ability to produce powerful cytotoxins with the same mechanism of action and structural similarity, such as Shiga toxins (Stx) from bacteria and ricin and related toxins from plants. Ricin is a potent toxic molecule known since 1888 when this name was coined for the proteinaceous and toxic substance derived from the seeds of *Ricinus communis *(castor beans), also capable of agglutinating erythrocytes [1]. Ricin is a bipartite exotoxin consisting of two disulfide-bonded chains: a single B-subunit (34 kDa) with galactose-binding properties and a single A-chain (32 kDa) endowed with the enzymatic activity [2]. Many glycoproteins and glycolipids present on the eukaryotic cell surface contain galactose residues that are specifically recognized by the lectin B chain, which allows binding to cells and facilitates endocytosis. Thus, the cytotoxicity of ricin is rather non-specific.

The first demonstration of the primary cellular function inhibited by the toxin A chain was obtained in neoplastic cells [3]: ricin strongly affected protein biosynthesis, while DNA synthesis was only slightly impaired, and the uptake of amino acids and RNA synthesis were spared. This result was confirmed shortly afterwards in the rabbit reticulocyte cell-free protein synthesis system, in which very small amounts of ricin completely inhibited translation [4]. The latter data were obtained by using whole ricin, *i.e.*, the two disulfide-linked A and B polypeptide chains. The reduction of the disulfide bridge was found to induce opposite effects on the action of ricin on cell-free systems and on mice [5]. The inhibitory effect on cell-free translation *in vitro* was markedly enhanced by the treatment, whereas the toxic effect on mice was strongly reduced, indicating different biological functions for the two chains, *i.e.*, the requirement of the lectinic B-chain to mediate entry of the toxin into cells, and of the free A chain to inhibit protein synthesis. In 1973, the ribosome [6] and more precisely the 60S subunit [7] were identified as the macromolecular targets of the A chain of ricin within the protein synthesis machinery. It was subsequently found that the damaged ribosomes were unable to bind elongation factors [8,9], thus pinpointing the functional ribosomal impairment. The calculation of the kinetic constants of ribosome inactivation by ricin A chain (*K*_m_ = 0.2 µM, *K*_cat_ = 1400 ribosomes modified/min) produced final evidence for the enzymatic action of the toxin [10].

The specific enzymatic activity of ricin on ribosomes was elucidated after more than a decade by Endo and collaborators [11,12] who showed that treatment of rat liver ribosomes with ricin A chain induced removal of adenine in position A-4324, by specific cleavage of the bond linking the purine to ribose (*N*-glycosidic bond). This specific residue is located in a stem-loop region of the 28S rRNA, which is one of the most strongly conserved rRNA sequence (5′-AGUACG**A**GAGGA-3′ in bold the adenine removed by ricin), present in almost all living species, from bacteria to eukaryotes [13]. Elegant footprinting experiments [14] have clearly demonstrated that this sequence is part of the recognition binding sites (consensus sequence) for prokaryotic (EF-Tu, EF-G) and eukaryotic(eEF-1, eEF-2) elongation factors. Since many different proteins capable of inactivating ribosomes were isolated in different plant species, they were systematically assayed for the ability to remove adenine from eukaryotic ribosomes. All of them shared the same enzymatic activity [15]; henceforth, these enzymes were officially classified as rRNA *N*-glycosidases (EC 3.2.2.22.) and the denomination ribosome-inactivating proteins (RIPs) was coined. 

**Figure 1:**
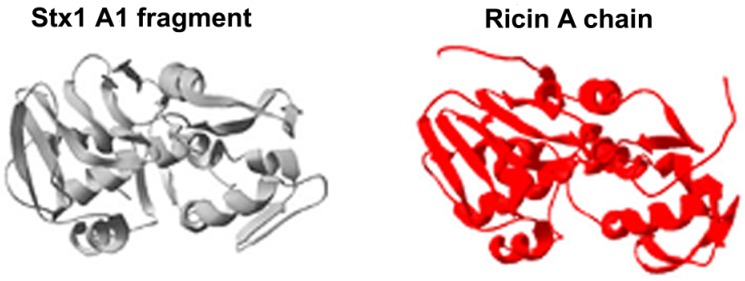
Folding of the A chain of ricin, compared to the A1 fragment from Stx [30] (with permission from Elsevier).

In the meantime, Shiga toxin from *Shigella dysenteriae*, the etiological agent of bacillary dysentery, was found to be a potent inhibitor of protein biosynthesis in cells [16,17,18] and in cell-free systems [19]. A breakthrough in the field was the purification to homogeneity of microgram amounts of the bacterial toxin obtained in 1980 [20], and evidence of the selective enzymatic inactivation of the 60S eukaryotic ribosomal subunit by purified Shiga toxin [21]. A few years later, the same Japanese researcher who had discovered the molecular mechanism of action of ricin, produced evidence that Shiga toxin and the related Shiga toxin 1 (Stx1) and Shiga toxin 2 (Stx2) (see below) removed the same adenine as did ricin from 28S rRNA [22]. Thus, bacteria and plants have probably converged during evolution to produce toxic molecules acting on this Achilles’ heel of ribosomes. However, ribosomal RNA is not the sole intracellular substrate of these toxins. All plant RIPs and Stx tested have been found to remove multiple adenines from DNA *in vitro* [23,24,25]. The early nuclear DNA damage observed in human endothelial cells challenged with ricin and Stx might be relevant to the mechanism of intoxication and constitutes a further convergence for plant and bacterial RIPs [26,27]. These common enzymatic behaviors are justified by the structural similarity between ricin and Shiga toxin deduced from their crystal structures [28,29]. The A region of Shiga toxin harboring the active site (A1 fragment, see below) and the A chain of ricin show high levels of similarity, whereas the B chains of these toxins are quite different [28,29]. Indeed, the folds of these A subunit regions that have 149 structural equivalent residues (23% of them identical) are nearly superimposable (Figure 1) [28,29].

Seven of the invariant residues (Tyr-77, Val-78, Ser-112, Tyr-114, Glu-167, Ala-168, Arg-170, and Trp-203; numbers as in Shiga toxin) are present in the active site cleft and some of them interact directly with adenine forming hydrogen bonds (Val-78, Ser-112, Tyr-114, and Arg-170; numbers as above) allowing the recognition of the substrate [28,31,32,33]. Thus, ricin and Stx share structural similarities, the same enzymatic activity and different binding specificity for eukaryotic cells.

## 2. Pro-Inflammatory Cytokines Produced by Eukaryotic Cells in Response to Ricin and Stx

Many authors have demonstrated independently that treatment of different cell types with ricin induces a specific response leading to increased mRNA levels and protein expression of pro-inflammatory cytokines such as TNF-α (tumor necrosis factor-α), IL-1β (interleukin-1β), IL-8 [34,35,36,37]. Such response patterns might contribute to ricin intoxication through the recruitment of inflammatory cells in the target organs. A relationship between the enzymatic activity of ricin and gene up-regulating effects might be envisaged in view of the fact that sequence-specific 28S rRNA injuries induced by ricin trigger the activation of the stress-activated protein kinases JNK (Jun *N*-terminal kinase) and p38 MAP (p38 mitogen activated protein) [38,39] which are implicated in the up-regulation of these pro-inflammatory genes [35,36,39,40]. In fact, it is recognized that 28S rRNA constitutes a ribo-sensor involved in the response of cells to various other stimuli converging on ribosomes, such as UV irradiation and antibiotics [38,39]. 

It should be noted that very similar gene up-regulating effects have been observed in the case of Stx acting on intestinal epithelial cells, endothelial cells and monocytes [35,37,41,42,43] and it has thus been suggested that these pro-inflammatory molecules are involved in the pathogenesis of human diseases, such as hemolytic uremic syndrome (HUS, see below), consequent to infections by bacteria producing Stx [44]. In particular, chemokines such as IL-8 and MCP-1 (monocyte chemoattractant protein-1) [42] and cell adhesion molecules [45] are up-regulated in endothelial cells intoxicated with Stx. These interesting observations were extended by microarray experiments showing that only 24 or 25 human genes, out of thousands tested, appeared to be up-regulated by Stx2 and Stx1, respectively [46]. Moreover, these genes encode mediators associated with inflammation, probably contributing to the recruitment of leukocytes in target organs of diseased patients [46]. Finally, Stx2 showed stronger up-regulating effects than Stx1 [27,46], and this might provide the molecular explanation of the epidemiologic association between Stx2-producing *Escherichia coli *strains and Hemolytic Uremic Syndrome (HUS) [47,48]. Also, in the case of Stx, the enzymatic activity of the A chain seems to be involved in triggering the up-regulating effects via activation of the ribotoxic stress, since treatment of cells with non-toxic Stx mutant [49] or challenge with monoclonal antibodies to Stx receptors or with purified Stx B chains [50] did not elicit those effects. However, identical impairments of translation and overlapping time courses of ribotoxic stress were observed for both Stx variants acting on human endothelial cells, so further regulations are probably operative in intoxicated cells, which enhance the Stx2-induced up-regulating effects [27]. 

**Figure 2:**
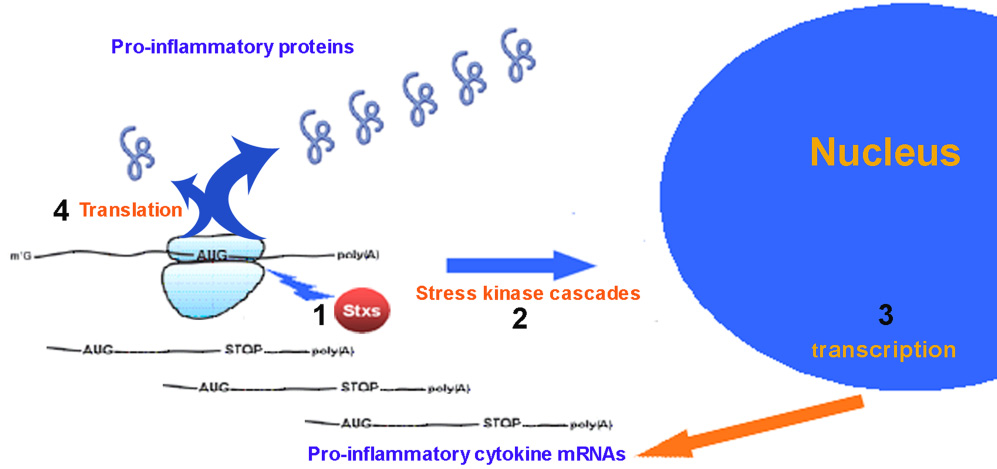
Relationship between ribotoxic stress and expression of pro-inflammatory cytokines involved in the pathogenesis of HUS. Ribotoxic stress imposed by Stx (point 1) triggers the activation of stress kinase pathways (point 2) culminating in the transcription of several pro-inflammatory cytokine mRNAs (point 3). The fate of these messengers is strictly related to the level of Stx-induced ribosomal damage that may affect the translation into proteins (point 4) of these important mediators of inflammatory damage in HUS.

In conclusion, as shown in the scheme (Figure 2), the main molecular link between cytokine expression and the enzymatic action of ricin and Stx is the ribosome, since the specific damage of ribosomal 28S RNA induced by the toxins is indispensable and sufficient to activate stress kinase cascades [27,35,36,39,40,51]. This, in turn, induces the nuclear transcription of pro-inflammatory genes via activation of appropriate transcription factors, such as NF-kB (nuclear factor kappa B) and AP-1 (activator protein-1) [42,50]. However, the same ribosomal damage might interfere with the translation into proteins of the pro-inflammatory cytokine mRNAs. Therefore, the relationship between cytokine expression and protein synthesis inhibition is quite complex, as a balance needs to be reached between the ribotoxic stress that activates transcription and the inhibition of protein synthesis that would in turn impair the expression. For this reason, the amount of pro-inflammatory mediators released by intoxicated cells progressively increases as the concentrations of ricin or Stx approach the IC_50 _on protein synthesis, whereas when toxin concentrations rise over the IC_50_, the production of pro-inflammatory substances is dampened or even blocked, due to an almost total impairment of translation [27,37]. This is the paradox of two potent inhibitors of protein synthesis, which, however, induce the regulated and specific expression of proteins. 

## 3. Role of Stx in the Pathogenesis of HUS

Several excellent reviews have outlined the main steps of the pathogenesis of STEC (Stx-producing *Escherichia coli*) infections as follows. The present review is aimed at showing the role of neutrophils/Stx interaction in this context, with an attempt to account for the conflicting results. 

STEC constitute a public health concern because they can cause food- or waterborne illness, whose clinical spectrum in humans ranges from mild to more severe manifestations, such as diarrhea, hemorrhagic colitis and HUS [52,53,54,55]. Most HUS cases are the life-threatening *sequela* of STEC intestinal infections [52,53,54,55] and the toxins produced by these bacteria have a causative role in the pathogenesis of HUS [56]. This syndrome represents the most common cause of acute renal failure in early childhood, being also characterized by thrombocytopenia and microangiopathic hemolytic anemia [54,57,58]. STEC strains elaborate Stx1 and Stx2: the two main antigenically distinct variants of Shiga toxin produced by *Shigella*[ 56]. Stx1 differs from Shiga toxin by a single amino acid in the A chain [59], whereas Stx2 has less than 60% similarity with Shiga toxin [56,58]. The toxins are composed of a B chain (7.7 kDa) organized in a doughnut-like pentamer which enables binding to the specific cellular receptor, the glycolipid globotriaosylceramide (Gb3Cer) [56,58]. The single A chain (32 kDa) is responsible for the enzymatic activity whose maximal expression is dependent on the proteolytic cleavage induced by the cellular protease furin and by reduction of the disulphide bond connecting the two resulting fragments A1 (28 kDa) and A2 (4 kDa) [56,58]. While the latter fragment dips its COOH-terminus into the center of the B-pentameric ring, non-covalently connecting the different chains of the holotoxin [28], the A1 fragment possesses the enzymatic deadenylating activity and directly induces intracellular injuries [56,58]. In humans, only a restricted subset of cells harboring Gb3Cer are targeted by Stx. Microvascular endothelial cells in the kidney, the intestine and the brain express Gb3Cer and, even though the toxins also damage mesangial cells and tubular epithelial cells in the kidney, endothelial injury is considered the main pathogenic event in HUS [56,60]. Targeted endothelial and non-endothelial cells showed a broad spectrum of responses including the production of pro-inflammatory cytokines involved in HUS pathogenesis (as reviewed above) [60,61] and the triggering of the apoptotic program [62,63,64]. In the latter case, multiple pathways seem involved, such as Stx-mediated ribotoxic stress, Stx-induced DNA damage, B chain interaction with cell receptors and endoplasmic reticulum stress response activated by altered host proteins or unfolded A1 fragments in intoxicated cells, with consequent imbalance of pro- and anti-apoptotic Bcl-2 (B-cell lymphoma-2) proteins [62,63,64]. 

After STEC ingestion and a short incubation time (approximately 3–5 days), the initial symptoms (abdominal cramps and diarrhea) are manifested by patients [52,53,56,57]. These symptoms are probably not related to the action of Stx, but are rather caused by the characteristic mechanism of adhesion of these *E. coli* strains to the gut mucosa [65,66]. Indeed, these bacteria possess the Type III secretion system encoded on the LEE (locus for enterocyte effacement) pathogenicity island. This molecular syringe is responsible for the injection in the enterocytes of LEE and non LEE-encoded effectors allowing the bacterial colonization of the mucosal epithelial cells of the bowel [67]. This results in the intimate adhesion of STEC to the gut mucosa and in the characteristic “attaching and effacing” cytopathology [56,60]. The intestinal brush border is disrupted and the enterocytes in intimate contact with the bacteria lose microvilli and accumulate cytoskeletal components forming structures known as pedestals beneath the cell surface [56,60]. This complex series of events might be sufficient to produce non-bloody diarrhea, without necessarily invoking a role for Stx. It should be noted that several STEC serotypes that are not associated with HUS are LEE positive [67], thus the virulence factors causally involved in the initial symptoms of STEC infections are not sufficient to trigger HUS. 

Stx produced by STEC are encoded by genes located on genomes of bacteriophages that occur in these bacteria as prophages. Effective expression of toxin genes requires prophage induction and subsequent lytic development of the bacteriophage [68]. When this happens, adherent bacteria produce Stx that are released in the intestinal lumen. The non-invasive bacteria are confined to the gut, whereas their toxins can translocate across the polarized epithelium of the bowel into the circulation. Although a contrary report has been recently published [69], a large body of evidence indicates that human intestinal epithelial cells do not express Gb3Cer nor other Stx receptors as reviewed in [70]. In contrast, human colon carcinoma cell lines showed high Gb3Cer expression and this renders Stx suitable for targeted cancer therapy [71,72]. Moreover, sophisticated methods such as Stx TLC (thin-layer chromatography) overlay assays followed by MALDI (matrix-assisted laser desorption/ionization) mass spectrometry confirmed only scant expression of Gb3Cer in surgical specimens of human normal colon [70,73]. However, this expression is not restricted to enterocytes, since minor cellular components expressing Gb3Cer are present in the human gut mucosa (Paneth cells) and might be involved in Stx translocation across the intestinal epithelium [70]. It is worth noting that Stx can reach the gut *lamina propria* also by Gb3Cer-independent mechanisms: (i) uptake of toxins induced by the macropinocytotic activity of intestinal epithelial cells [74], (ii) disruption of intestinal epithelial tight junctions induced by STEC/enterocyte interactions [70] or, at a later time, by the opposite transmigration of polymorphonuclear leukocytes (PMN) in the inflamed intestine [75,76]. Independently of the mode of translocation through the gut epithelial barrier, having arrived in the *lamina propria*, some Stx molecules are trapped by the Gb3Cer receptors present in the endothelial cells lining the microvasculature of the gut [70,73]. Intoxication of these cells could explain the typical histopathological changes observed in the gut: hemorrhage and edema of the *lamina propria* with focal areas of necrosis [60,61,77,78]. This scenario also explains the bloody-diarrhea experienced by approximately one-third of STEC-infected patients who develop hemorrhagic colitis [52,53,56,57]. 

About a week after the onset of diarrhea, HUS occurs in a variable percentage of patients (10–25%) [52,53,56,57]. During this phase, the Stx molecules escaping the binding to intestinal endothelia reach the Gb3Cer-containing endothelial cells of the microvasculature of the brain and the kidney. Under normal conditions, endothelia are not adhesive for platelets and the prevalent behavior is thromboresistance. However, in the presence of Stx these properties dramatically change, resulting in endothelial dysfunction. Endothelial cells in renal glomeruli alter their adhesion properties and produce pro-inflammatory cytokines and chemokines [60,61,78]. The damage to endothelium and the induction of pro-inflammatory mediators triggered by Stx is causally related to microvascular thrombosis in the kidney [60,61,78]. Histologically, HUS is characterized by widespread thrombotic microvascular lesions in the renal glomeruli, the gastrointestinal tract and other organs, such as the brain [56,77,78,79]. The examination of glomeruli from patients with HUS revealed capillary wall thickening, swelling and detachment of endothelial cells from the basement membrane exposing sub-endothelial matrix [56,77,78,79]. Under these conditions, circulating platelets promptly adhere to the subendothelial matrix, thus becoming activated, and bind to one another (aggregation) with the deposition of fibrin. The widespread formation of glomerular microthrombi by narrowing the vascular lumen might compromise blood supply determining acute renal failure, the main hallmark of HUS [56,60,78]. 

The strong reduction in circulating platelets observed in HUS is believed to be caused by both the direct and indirect effects of toxins [60,78,80]. Although the matter has been widely debated, several lines of evidence indicate that Stx, besides activated platelets [81], can directly interact with resting platelets leading to their activation, changes in ultramorphology, increased fibrinogen binding activity and aggregation [82]. Platelet aggregates are removed by the reticuloendothelial system, hence reducing the number of circulating thrombocytes. In addition, the massive engagement of platelets in the damaged microvasculature of the kidney might further contribute to thrombocytopenia [60,78,80]. It has been recently shown that Stx, in cooperation with bacterial lipopolysaccharide (LPS), induced the formation of aggregates between leukocytes and platelets, leading to a release of tissue factor–bearing microparticles. Complement activation on these complexes and on microparticles might indicate their role in the prothrombotic state occurring in HUS [83,84]. 

Finally, the hemolytic anemia, the third component of the triad that characterizes HUS, is assumed to be the consequence of the mechanical injury induced by microthrombi to the erythrocytes passing through narrowed renal capillaries. This process results in the formation of schistocytes and helmet cells and their sequestration by the reticuloendothelial system [56,60,78]. Thus, Stx are critical virulence factors in the development of HUS, as well as in triggering hemorrhagic colitis. However, only a few STEC serotypes, in particular O157, O26, O111, O103 and O145, are associated with HUS, suggesting that a critical combination of bacterial factors must cooperate with toxins in triggering HUS [67,85]. On the other hand, ricin-injected mice and rats develop the hallmarks of HUS [40,86,87] and the latter syndrome was noted as an adverse effect during treatment of cancer patients with ricin-containing immunotoxins [88]. This further corroborates the view that ricin and Stx are also analogous in inducing peculiar and distinctive afflictions in humans. Obviously, the complex series of bacteria/host interactions occurring during the natural course of STEC infections that allow the bacterial toxin to reach circulation in humans is not entirely replicated by the experimental or therapeutic injection of the whole plant toxin or of its enzymatic moiety in animals or patients, respectively. As previously stated, these toxins showed structural similarity and functional identity in their A chains, rather than in B chains. This is in keeping with the idea that some crucial steps in the development of HUS involve the active chains, whose role could not be simply related to their enzymatic activity. 

## 4. Transport of Stx in Blood and the Role of Stx/PMN Interactions

Since systemic toxemia is considered to be central to the genesis of HUS, many authors tried to demonstrate the simplest hypothesis to explain why STEC confined to the gut might determine kidney and brain intoxication, *i.e.*, the movement of free toxins in blood. However, Stx was never detected in the sera of HUS patients with concomitant detectable fecal toxin [52,89,90]. In the fundamental study by Karmali’s group [52] in which the authors provided the first demonstration of the association between HUS and STEC infections, sera from 27 STEC-infected patients with overt HUS were tested by Vero cell cytotoxicity assay with negative results, even though 50% of them showed free fecal Stx and sampling times for feces and serum were basically the same. Two subsequent studies confirmed the absence of Stx in sera from 37 (Vero cell cytotoxicity assay) [89] and 34 (human umbilical vein endothelial cell radioactive protein synthesis assay) [90] STEC-infected HUS patients. In the last study, it was also demonstrated that the cytotoxic activities of Stx1 and Stx2 added to blood from healthy donors were completely recovered after the preparation of sera and did not change after prolonged incubation at room temperature, nor after repeated freezing and thawing cycles. Although blood from patients with fully developed HUS did not contain detectable amounts of free Stx, a fleeting appearance of the toxins in blood followed by a rapid clearance before the onset of renal failure cannot be ruled out. 

The second hypothesis to explain the delivery of Stx is the shuttling by macromolecular or cellular blood components. In theory, it would seem unnecessary for toxins entering the circulation to initially find a receptor on circulating cells or a binding site on a macromolecule rather than going directly to the receptors of target cells in kidney and brain. However, there is evidence for this idea that Stx interact with various blood cells. *In vitro* experiments produced evidence of Stx binding to erythrocytes [91], monocytes [41,92], platelets [81,82,93] and lymphocytes [94]. 

In the human P group system the antigen Gb3Cer, namely P^k^, is present on the very rare Pk1 and Pk2 phenotypes, rendering the corresponding erythrocytes capable of binding Stx. In addition, the erythrocytes belonging to the more common P1 (80–95% of the population) and P2 (most of the remaining) groups possess small amounts of the Gb3Cer/P^k^ antigen. Indeed, Stx were found to bind to red cells having P1 and P2 phenotypes [91]. However, direct binding of Stx to erythrocytes was not observed in cases of human STEC-induced disease. 

Human monocytes express small amounts of Stx receptors that appeared related to, but different from, the Gb3Cer lipoforms present on endothelial cells [41]. The number of binding sites on monocytes might be enhanced by activation after treatment with bacterial endotoxins [41]. The monocyte/Stx interactions induce the secretion of the pro-inflammatory mediators TNF-α and IL-1β [41]. The latter by up-regulating Gb3Cer receptor expression enhance the sensitivity of endothelial cells to the toxins [95,96,97]. However, the passage of Stx from monocytes to endothelial cells does not occur [92], since both cells harbor receptors with similar affinities [41]. 

Platelets bind Stx through Gb3Cer and a different glycosphingolipid, namely band 0.03, but the distribution of these platelet receptors in humans is quite heterogeneous [93]. Moreover, Gb3Cer expression on resting platelets is very low [81], and platelets internalize Stx within 2 h, with consequences such as aggregation, changes in ultramorphology and increased fibrinogen binding capacity [82]. As stated above, it is reasonable to conclude that direct binding of the toxins to platelets might be involved in the prothrombotic state, leading to the thrombocytopenia seen in HUS, rather than in the passive transfer of Stx to other body sites. 

Gb3Cer is also expressed on the surface of a narrow range of committed B lymphocytes present in germinal centers and on the corresponding B cell lymphomas, such as Burkitt lymphoma [98]. This subset of human B lymphocytes is susceptible to the cytotoxic action of Stx [94] and this would tend to rule out the hypothesis that B lymphocytes are carriers of Stx *in vivo*. 

A breakthrough in this scenario was the discovery of the role of PMN in binding Stx and in transferring them to endothelia [99]. PMN have been considered of prime pathological importance in HUS for different reasons: a high neutrophil count at presentation is a typical finding in patients with HUS, and this was found to be strictly related to an adverse outcome in the infected children [100,101,102]. Neutrophils in HUS patients are activated and degranulated as inferred by the presence in the blood of patients of high levels of elastase, a protease present in azurophil granules of PMN [103], and also by the direct observation of the functional state of PMN in HUS patients [104,105,106]. PMN degranulation and activation also correlate with poor prognosis [104,107] and PMN from HUS patients are more adhesive to cultured endothelial cells than to control cells and are directly implicated in experimental endothelial injuries *in vitro* [108]. Since PMN emerged as main actors in the pathogenesis of HUS, it was no surprise that they could bind Stx *in vitro *[99]. The authors of this pivotal paper led by Victor van Hinsbergh (corresponding author) and Leo Monnens reported impressive evidence of Stx binding to PMN. They found by direct flow cytometric analysis and direct immunohistological studies that the addition of fluorescent Stx1 to human whole blood or to purified PMN, erythrocytes, monocytes, platelets and lipoproteins resulted in specific binding to PMN and negligible binding to other blood components. Thus, for the first time, a careful, comparative quantitative analysis was performed. Moreover, by using iodinated Stx1, they calculated, using Scatchard plot analysis, the dissociation constant (*K*_d_ = 10^–8 ^M) of the interaction PMN/Stx1 and the number of binding sites per cell (2.1 × 10^5^). Furthermore, the lack of the internalization of the toxic radioactive cargo by PMN was clearly demonstrated. This latter behavior, together with the 10-fold lower affinity of Stx for PMN compared to Gb3Cer (*K*_d _= 10^–9^ M), led the authors to hypothesize a transfer of the toxic ligand from these leukocytes to the glomeruli of the patients. The transfer was experimentally demonstrated *in vitro* after incubation of PMN carrying Stx with human glomerular vascular endothelial cells (i) with fluorescent toxin by flow cytometric analysis, and (ii) with native toxin by cytotoxicity and protein synthesis radioactive assays. An important point addressed by the authors was the demonstration that the transfer of the toxin or the appearance of considerable effects on endothelial cells (toxicity, inhibition of translation) occurred after stimulation with TNF-α, a treatment known to enhance the expression of Gb3Cer on these cells [95,96,97]. However, a few years later, some of these authors led by Leo Monnens reversed their position, by claiming that the binding of Stx to PMN was not specific and, basically, re-interpreting their previous results as artefacts [109]. On the one hand, this dampened the enthusiasm for this interesting explanation of the mode of delivery of Stx from the *lamina propria* of the bowel to the endothelia of brain and kidney and, on the other hand, it stimulated scrutiny and intense debate on this topic. At the present time, there is no consensus in the literature for a variety of sound reasons along with some preconceptions. The sound reasons are based on the conflicting results obtained by different groups over the last decade on the binding of Stx to PMN and on the functional consequences of such a binding. However, the preconceptions that Stx/PMN interactions are unreliable or non-specific or of little biological significance have been widely diffused, leading to some ostracism by the scientific community. Leaving out the publications of my group, many sound articles have been written on this topic with clever hypotheses, clear-cut experimental plans involving well-conducted and technically sound experiments, leading to well-founded deductions that, unfortunately, have led to opposite conclusions. This does not necessarily mean that the results are unreliable or flawed; rather, it should prompt the scientific community to investigate the matter in more detail to reveal the explanation.

In this review, first the papers in which direct evidence of Stx binding to PMN has been sought will be considered. Then the articles reporting indirect positive or negative evidence of such a binding will be reviewed. It is worth noting that the first questioned paper published on Blood [99] was never retracted by the corresponding author. To the best of my knowledge, no item of that paper was proven to be fraudulent or derived from incorrect experiments or misleading interpretations. It should also be noted that very similar experimental techniques had been employed by the same scientists who authored the Blood paper demonstrating the binding of Stx to monocytes without stimulating any further criticism [41]. Thus, the results reported on the Blood paper should be considered as evidence supporting the reliability of Stx/PMN interactions, whereas the second paper [109] represents opposing evidence. In the latter study, the binding of iodinated Stx1 to isolated human PMN from seven donors was shown to be ineffective. Moreover, the authors demonstrated the absence of PMN-binding activity of the iodinated B subunits added to whole human blood (7 donors) or injected in mice (9 animals). In both cases, most of the radioactivity was found in the plasma.

There appears to be a real dichotomy on this topic. The binding of Stx to human PMN *in vitro* has been demonstrated experimentally by other three different groups [83,110,111,112,113,114], even though Karpman and colleagues found minimal binding of the toxins to neutrophils [83]. This would be expected since the authors challenged whole blood with 10^–12^ M Stx2 concentrations with respect to the 10^–9^ M concentrations required to saturate PMN receptors [99,114]. The positive results on the ability of Stx to bind human PMN were obtained with methods based on fluorescent toxins or fluorescent antibodies (indirect and direct cytofluorimetric analysis, immunofluorescence) or radioactive-labeled toxins and by employing whole blood or isolated PMN. With indirect cytofluorimetric analysis [105] and ELISA [115], employing whole human blood and/or isolated PMN, two different groups failed to detect such a binding. The matter remains controversial. It is also not established whether Stx binding to PMN can be inferred by the presence of functional modifications in leukocytes after the putative interaction with toxins. Several groups found no activation of PMN in terms of expression of degranulation markers or integrins on the plasmatic membrane [105,109,116], superoxide production [109,117,118] and elastase release [117] in resting or primed PMN. By contrast, a degranulation response with subsequent hyporesponsiveness to a second activating (IL-8) stimulus [114] and a dose-dependent induction of superoxide with concomitant reduced ability for phagocytosis and reduced response to PMA (phorbol myristate acetate) was observed after incubation of PMN with Stx [116]. With respect to the indirect evidence, a delaying effect of Stx on the extent of spontaneous neutrophil apoptosis was demonstrated first by Liu and colleagues [119] by flow cytometry up to 48 h. Again, this result was followed by a confirmatory paper in which the PMN-apoptotic delay induced by the toxins was assessed by morphological examination of nucleus shape (10 h and 24 h), measure of caspase 3 activity (5 h) and flow cytometric analysis (48 h) [112]. However, contradictory results [115,116] obtained with similar cytofluorimetric techniques at 20 h have been published.

In reviewing all the papers dealing with direct and indirect evidence of Stx/PMN interactions, it would appear that positive or negative results are not related to the type of toxin variants (Stx1, Stx2) used in the different studies. Conversely, it is worth noting that these apparently conflicting papers exhibit an intrinsic coherence, *i.e.*, the papers in which evidence has been presented of the binding of the toxins to PMN also contained clear indications of functional modifications of leukocytes. Contrariwise, those reporting negative results on binding failed to show functional consequences. Strikingly, when PMN were studied in patients with HUS, they were found to be activated, degranulated and hyporesponsive to other stimuli and delayed in the apoptotic program [104,105,106]. Interestingly, the same PMN features observed *in vivo* were also noted by the authors who challenged PMN from healthy donors with Stx, obtaining positive results (see above). More importantly, those who sought the toxins bound on the surface of PMN in HUS patients, by means of indirect cytofluorimetric analysis, invariably found them (see below) [83,90,111,120,121]. This strongly suggests that Stx are directly responsible for the functional changes observed in the PMN from patients, since they are bound to their surface during the natural course of the disease. Other explanations of the state of PMN in patients are based on an early Stx-independent activation of PMN, related to the inflammatory status of the bowel and of the kidney. It can be argued that a massive liberation into the blood of activating pro-inflammatory cytokines from these body sites might activate circulating PMN, as happens in sepsis. However, incubation of PMN from healthy donors with plasma from HUS patients did not render these leukocytes activated, degranulated or hyporesponsive to other stimuli [105], nor were they resistant to apoptosis [104]. Moreover, although PMN from HUS patients showed delayed apoptosis and prolonged life-span [104], they would soon disappear from circulation, since the half-life of PMN in blood is about 6 to 7 h [122]. Indeed, every day a huge number of PMN (10^11 ^cells) enter the blood from bone marrow, replacing the cells that leave blood by transmigration. Defective chemotaxis [117] or impaired transmigration [114] of Stx-treated PMN have not been observed. In conclusion, it is unlikely that a single stimulus induces the stable activated state of PMN in patients. It is more likely that there is a continuous challenge from the long-lasting persistence of Stx on the membrane of PMN in HUS patients [120,121] (see below). Thus, the reasons behind the discrepancies obtained in the reports dealing with *in vitro* binding experiments with purified Stx need a further explanation. As suggested [110], the experimental procedures employed to isolate PMN after toxin challenge differ somewhat. Two of the groups reporting negative results [109,115] isolated cells and removed free unbound toxins by centrifugation on Ficoll layers or Mono-Poly resolving media, whereas mild isolation conditions assuring the minimal perturbation of the samples were employed by two groups which gave positive results [110,111,112,114]. The former treatments could have caused the detachment of Stx from PMN because of the low affinity of the Stx/PMN interaction, although it should be borne in mind that low-affinity binding does not mean a lack of specificity. Indeed, some experts in the field have failed to appreciate the relevance of the binding of Stx to a low-affinity receptor (*K*_d_ = 10^−8^ M), defining the weak binding to PMN as non-specific and of little biological significance. However, PMN possess other receptors capable of specific and low-affinity binding to several important molecules. For example, neutrophils during the phagocytosis of opsonised particles or the binding to immunocomplexes, are capable of interacting with the Fc portion of some IgG subclasses through different Fc receptors whose affinities largely differ depending on the specific function required: FcγRI/CD64 (*K*_d_ = 10^–9 ^M) also binds to monomeric IgG, whereas FcγRIIA/CD32a and FcγRIIIB/CD16b have lower affinity (*K*_d_ = 10^–7 ^M) [123,124,125]. These binding activities are not considered to be of little biological significance, rather, they are fundamental to the function of these leukocytes. Moreover, most of the papers confirming such a weak Stx/PMN binding also showed evidence of specific interactions, as the bound labeled (either fluorescent or radioactive) toxins were displayed by an excess of cold homologous Stx [99,112] or the functional consequences of the binding was dampened after heat-inactivation [112,116,119] or treatment with monoclonal antibodies to Stx [119]. Finally, PMN low-affinity binding might be advantageous in a model based on the transfer of the ligand to high affinity target receptors, such as endothelial Gb3Cer.

Another possible explanation for the discrepancies in the reviewed studies is that toxins prepared in different laboratories might be somewhat different. To further our understanding of this matter, Table 1 summarizes the different methods of purification of the toxins used in those papers, as well as the LPS content of each preparation. First, it is worth noting that the pivotal study demonstrating for the first time the binding to PMN [99] and the subsequent study in which some of the authors reversed their position [109] both employed Stx1 given by Dr. Karmali (Health, Toronto Canada). However, the batches of toxins were not the same (Leo Monnens, personal communication), with possible differences in the properties of Stx in the two different preparations. Second, although not all the papers reported the amounts of contaminating LPS in Stx preparations, endotoxin ranged from femtogram to picogram units/Stx µg (median 1.5 pg/µg, *n *= 4) in the positive studies, whereas it ranged from tens of picogram to ng/Stx µg (median 40 pg/µg, *n *= 3) in negative studies (Table 1). This indicates that contaminating LPS may not be responsible for the functional effects shown in Stx-treated PMN in the positive studies, since negative results have been obtained with higher amounts of contaminating endotoxin. On the other hand, the very low amount of LPS together with Stx in the positive reports renders unlikely a role for a binary Stx/LPS complex, since the calculated molar ratio (assuming molecular mass > 10^4^ Da for LPS and 68,000 Da for Stx) is extremely high. Third, and more importantly, five positive studies out of six reported short purification schemes in which Stx were resolved by different types of affinity chromatography (globotriose-fractogel, Synsorb pK, P1) reducing the purification procedure to one or a few steps. Conversely, with one exception, all the groups that obtained negative results purified the toxins by multistep methods (4-5 steps) or obtained them from commercial sources (Table 1). Only in one case was the toxin purified by single-step affinity chromatography (Table 1). 

**Table 1:** Synoptical analysis of Stx purification procedures from publications with conflicting results on Stx/PMN interactions.

Paper	Toxin	Purification scheme ^a^	LPS ^b^ pg/µg	Purification method
**Positive results**				
Brigotti *et al*., [112,114]	Stx1	AGl	<3	[126]
	Stx2	AS, AE, AP1	<3	[127] ^c^
Griener *et al*., [110]	Stx1	ASy	<0.077	[128]
	Stx2	ASy	<0.077	[128]
King *et al*., [116]	Stx1	AS, AP1	<3	[129]
Liu *et al*., [119]	Stx2	CE, HPLC	n.a.	[130] ^d^
	rStx2	Dr. Gondaira (Denka Seiken, Tokyo)	<0.001	n.d.
Stahl *et al*., [83]	Stx2	AP1^e^	n.a.	[131]
Te Loo *et al*., [99]	Stx1	Dr. Karmali (Health, Toronto, Canada)	n.d.	n.d.
**Negative results**				
Aoki *et al*., [118]	Stx1	AS, AE, Ch, HPLC	<2500	[132]
	Stx2	AS, AE, Ch, HPLC	<2500	[133]
Fernandez *et al*., [105]	Stx1	Dr. Juniki (Denka Seiken, Nigata, Japan)	<40	n.d.
	Stx2	Dr. Juniki (Denka Seiken, Nigata, Japan)	<40	n.d.
Flagler *et al*., [115]	Stx1	AS, AE, HA, AG, GF	<11	[115]
	Stx2	AS, AE, AP, AG, PS	<11	[115]
Geelen *et al*., [109]	Stx1	Dr. Karmali (Health, Toronto, Canada)	n.d.	n.d.
	Stx2	Toxin Technology, Sarasota FL, USA	n.d.	n.d.
Holle *et al*., [117]	Stx1	Prof. Lord (Warwick University, UK) AGl^ f^	n.d.	[126]
	Stx2	Toxin Technology, Sarasota FL, USA	n.d.	n.d.

^a ^Abbreviations: AE, anion exchange; AG, Affi-Gel blue; AGl, globotriose affinity chromatography; AP1, P1 affinity chromatography; AS, ammonium sulfate precipitation; ASy, Synsorb pK affinity chromatography; CE, cation exchange; Ch, chromatofocusing; HA, hydroxyapatite chromatography; HPLC, high-performance liquid chromatography; PS, Phenyl-Sepharose; n.a. not applicable since the LPS content was reported in volume and not per toxin amount; n.d. not done; ^b ^To compare data from different papers, the conversion from CSE potency of LPS to LPS amount was performed according to the ratio Eu/ng = 13; ^c ^Modified by [127]; ^d^ Modified by [130];^ e ^Dr. D. Karpman’s (Lund University, Lund, Sweden) and Dr. A. Kane’s (Tufts Medical Center, Boston, USA) personal communications;^ f ^Prof. M. Lord’s (University of Warwick, Coventry, UK) personal communication.

All the recognized methods listed in Table 1 seem sound and ensured purification to homogeneity and preservation of the enzymatic and toxic activity of Stx as directly assessed by the authors of the studies and/or as reported in the references covering the purification methods. Thus, the conclusions reached by the authors of the negative papers were not incorrect: fully-active toxins with no binding-activity for PMN. However, we recently made the serendipitous observation that it is possible to obtain fully toxic Stx1 lacking PMN binding activity [134]. By means of spectroscopic and fluorescence techniques, our group demonstrated that a partial unfolding of the toxin, with reduction of α-helix content and exposure of some hydrophobic Trp residues led to loss of PMN binding activity [134]. Interestingly, the enzymatic activity linked to the A chain and the Gb3Cer binding activity related to B chains were preserved and the unfolded toxin was found to be fully active in intoxicating human endothelial cells [134]. The loss in PMN-binding activity was partially reproduced by repeated freeze and thawing cycles, with conservation of toxic activity [134]. In conclusion, a reasonable explanation of the conflicting results regarding Stx/PMN interactions would be a partial conformational change of toxins prepared by more complicated and laborious multi-step purification methods. Obviously, these methods have been set-up to obtain pure Stx endowed with full cytotoxic or enzymatic activities, rather than adapted to the recovery of the then unknown PMN-binding activity. For this reason, we have proposed that purified toxins should also be assayed for correct folding, by inspecting circular dichroism and/or fluorescence emission spectra and their maxima. 

## 5. PMN Recognize the A Chain of Stx and Related Plant Toxins

Several lines of evidence indicate that the interactions between Stx and PMN are mediated by the A chain of the toxins. Competition experiments clearly showed that the PMN-binding domain on Stx is distinct from the Gb3Cer-binding domain. Indeed, the binding of Stx to human PMN *in vitro* is strongly increased by “DAISY” (a decavalent Gb3-related receptor analogue), which however inhibits Stx binding to Gb3Cer-expressing eukaryotic cells [110] and prevents Stx-induced lethal effects on mice [135]. Moreover, human serum amyloid P component (HuSAP) was found to bind specifically to Stx2 [136,137] and to inhibit the Gb3Cer-related cytotoxic activity of this toxin [136], protecting mice from its lethal effects [138]. Indeed, this putative competitor failed to inhibit and rather increased toxin binding to human PMN [110]. It should be noted that “DAISY” and HuSAP inhibited the bindingof Stx to murine [110] neutrophils endowed with Gb3Cer receptors also found in porcine [139] and ovine [140] granulocytes. Thus, Stx interact with murine, porcine and ovine PMN via Gb3Cer-dependent mechanisms, while Gb3Cer-independent mechanisms are operative in mediating Stx-binding to human PMN. This is consistent with the notion that human PMN do not possess the complete repertoire of enzymes necessary to synthesize glycosphingolipids of the globo-series, such as Gb3Cer [141]. Further support is provided by results obtained in the human myeloid leukemia cell HL-60 model for inducible cell differentiation [142,143]. These undifferentiated cells only possess trace levels of Gb3Cer [144] and do not bind Stx [112]. Conversely, differentiation of HL-60 to granulocytes by treatment with all-*trans*-retinoic acid, a condition which does not up-regulate Gb3Cer expression [145,146], enabled the cells to bind Stx [112]. The same behavior was observed with poorly differentiated immature human granulocytes obtained by healthy donors treated with G-CSF that, unlike mature PMN, do not bind Stx [112]. 

Taken together, these results indicate that mouse and human PMN differ in the expression and in the nature of Stx-binding receptors. The results obtained in mouse models on the delivery of toxins in blood [109,115] cannot, therefore, be considered representative of the toxic delivery pathways in HUS patients, even though they might be useful to mimic HUS renal damage and test the efficacy of new therapeutic approaches [147,148,149]. Moreover, in light of the evidence reported above, the negative results on PMN-binding obtained with isolated Stx B subunits in human blood can be considered a further proof of the involvement of A subunit [109]. 

Direct evidence of the binding to human PMN of Stx A subunit isolated by fast protein liquid chromatography has been presented [110], and this important contribution was the first report of the binding of the enzymatic chain of Stx to an eukaryotic cell. This behavior of the Stx A chains was confirmed by competition experiments showing that the A chain of ricin and two single-chain RIPs, gelonin (from the seeds of *Gelonium multiflorum*) and the non-glycosylated saporin S6 (from the seeds of *Saponaria officinalis*) impaired the binding of Stx to PMN, whereas diphtheria toxin was completely ineffective [113]. This is in keeping with the notion that A chains of ricin and Stx show structural similarities [28,29]. As such, the interaction of fluorescent ricin A chain with human PMN has been demonstrated directly by flow cytometric analysis: the binding is saturable, specifically inhibited by cold ricin A chain and the fluorescent subunit was not internalized by leukocytes [113] as well as Stx [99]. The calculation of the *K*_d_ value (10^−9^ M) and the number of binding sites of the ricin A chain/PMN interaction showed that the affinity of ricin A chain for PMN was 10-fold higher than that reported previously for Stx, while the number of binding sites per cell was of the same order of magnitude as those calculated for the Stx/PMN interaction [99,112]. Taken together, these observations indicate that Stx/PMN interactions are specific. Moreover, the fact that Stx and related plant toxins share, apart from structural similarities and identical enzymatic activity, a common receptor on PMN changes our perspective somewhat. It is not a simple interaction with eukaryotic cells to be targeted and killed by powerful toxins belonging to quite different biological kingdoms, but rather involvement of an actor of the innate immunity, the PMN, that recognize a class of dangerous potent toxins irrespective of the source. This is consistent with the known ability of these leukocytes to recognize molecular patterns common to different foreign molecules. The implications of such a binding in PMN of patients with HUS merit some discussion (see next paragraph).

Finally, other deductions on the PMN-binding site on Stx arise from the experiments reported above with unfolded toxin with impaired binding but fully active as toxic agent [134]. Unfolded Stx1 showed disruption of α-helix structures with exposure of Trp residues otherwise residing in hydrophobic environments in native toxin. From steady state and time resolved fluorescence studies and by reviewing functional and structural data, it is possible to identify the A chain moieties close to Trp203 and located in the A1 fragment as the region recognized by PMN. As stated above, the A1 fragment is the portion of Stx which possesses highly similarity with ricin A chain in primary sequence and secondary structure (Figure 1). In conclusion, the presence of a new binding site for PMN in the Stx A subunit, besides the enzymatic-active site and the well-known Gb3Cer-binding sites in the B subunits, represents a novel property of these well-characterized bacterial toxins. 

## 6. Transfer of Stx from PMN to Gb3Cer-Expressing Cells

The idea of circulating PMN as toxin carriers was first proposed by [99] based on experiments in which co-incubation of human glomerular microvascular endothelial cells with PMN carrying Stx resulted in the transfer of the toxin to the former cells. The data were obtained with fluorescent toxin, by monitoring the transfer by flow cytometry (fluorescent staining of 30% of endothelial cells and disappearance of fluorescence on PMN), and with native toxin, by measuring Stx-induced cytotoxicity and translation inhibition in targeted cells. The transfer was confirmed in similar experiments performed with PMN carrying fluorescent Stx and Ramos cells which express Gb3Cer receptors [110]. However, these important data were obtained in experimental models that did not reproduce the physiological mode of interaction of circulating PMN with endothelia. These interactions mainly occur in post-capillary venules, whereas the renal glomerulus is one of the few sites in which PMN recruitment occurs in capillaries [150]. Although PMN travelling in capillaries are close to the inner surface of the vessels, their interactions with endothelial cells are accidental and scanty in normally flowing blood, since they mainly occur during leukocyte transmigration in the presence of chemoattractants. By using an experimental model consisting of a confluent monolayer of human umbilical vein endothelial cells through which endotoxin-free PMN transmigrate, recruited by IL-8, it has been demonstrated that PMN diapedesis is not impaired by the presence of Stx on their membrane, even under saturating conditions [114]. While non-transmigrating PMN transferred only low amounts of toxins, during 2 h-transmigration Stx passed from PMN to endothelial cells, as shown by the disappearance of unlabeled or radioactive toxins from transmigrated PMN and by the concomitant radioactive labeling of the endothelium [114]. After overnight post-incubation, endothelial cells became intoxicated as assessed by the extent of translation inhibition which, in turn, depended on the number of transmigrating leukocytes carrying Stx. The addition of monoclonal antibodies to Stx during transmigration prevented endothelial cell intoxication, demonstrating the specificity of such a transfer. 

It should be noted that the observations obtained in *in vitro* experimental models of pathogenesis, although controlled and accurate, need to be validated in animal models and directly observed in patients, before concluding that they are really operative during the natural course of the disease. Thus, the demonstrated transfer of Stx from circulating cells to endothelia *in vitro* is still a suggestive hypothesis. The baboon model may constitute a relevant test, since these animals, unlike other animal models such as mice, have probably the same expression of Stx-receptors on PMN as humans. However, an improved animal model using baboons with intestinal infection by STEC would more accurately reproduce the course of the human condition during the passage of freshly produced toxins through the epithelial barrier into the circulation. 

## 7. Where Do PMN Bind Stx During the Natural Course of Disease?

This can be studied by analyzing the affinity properties of the toxins with respect to PMN and/or by reviewing the notions on the presence of Stx in the blood of patients (see next paragraph). The number of Stx or ricin A chain receptors on PMN is approximately 2 × 10^5^[99,113], accounting for the presence of about 1 fmol of toxins on 3000 saturated PMN in 1 µL of blood. However, the *K*_d_ of the toxin/PMN interactions are quite different in the case of the plant (10^–9^ M) or the bacterial toxin (10^–8^ M). The one order of magnitude difference means that relevant amounts of ricin A chain molecules are captured by PMN when they are challenged with toxin concentrations equal to or below the *K*_d_ (≤1 nM, Table 2) and that the amount of ricin A chain bound to PMN at full saturation of receptors is in equilibrium with a 10-fold excess of free ricin A chain (Table 2). In the case of Stx, however, the toxin saturating PMN receptor is in equilibrium with a 100-fold concentration of free toxin (Table 2). Moreover, free toxins largely exceed bound toxin (10–20 fold) at Stx concentrations equal to or lower than the *K*_d_(≤10 nM, Table 2). 

**Table 2:** Theoretical analysis of the relationship between saturation of PMN receptors, free toxins and PMN-bound toxins.

Total toxin (nM)	Stx1 ^a^			Ricin A chain ^a^		
	PMN receptor saturation (%)	Free toxin (%)	Bound toxin (%)	PMN receptor saturation (%)	Free toxin (%)	Bound toxin (%)
100	90.83	99.09	0.91	-	-	-
10	48.75	95.12	4.88	90.10	90.99	9.01
1	8.39	91.61	8.39	38.20	61.80	38.20
0.1	0.90	90.98	9.02	4.88	51.25	48.75
0.01	0.09	90.92	9.08	0.50	50.12	49.88
0.001	0.01	90.91	9.09	0.05	50.01	49.99

^a^ The data have been calculated according to the parameters obtained by Scatchard plot on the PMN/ricin A chain (*K*_d_ = 10^–9^ M; binding sites = 2 × 10^5^) [113]; or PMN/Stx1 (*K*_d_ = 10^–8^ M; binding sites = 2 × 10^5^) [99,112] interactions assuming the presence of 3000 PMN/µL of blood.

If Stx/PMN interactions are not perturbed *in vivo* by other factors and assuming that PMN encounter Stx in blood, this means that it is not possible to detect PMN-bound Stx in the absence of free toxins in circulation. This is in contrast with the clinical observations in patients with HUS, which indicate the presence of Stx on PMN in the absence of free toxin. In fact what happens in the *lamina propria* of the gut during the translocation of Stx in blood is complicated by the contemporaneous presence of resident (gut endothelial cells) and circulating (monocyte and platelets) cells harboring high-affinity Gb3Cer receptors. The areas of the bowel colonized by STEC are rather restricted with respect to the surface of gut endothelia. It is likely that Stx concentrations may be quite elevated, and probably sufficient to engage circulating PMN in the intestinal vessels supplying those areas of the bowel where toxin translocation occurs. Moreover, the concomitant inflammatory process of the gut might slow blood flow (stasis) increasing the time of engagement of PMN with toxins. In contrast, non-activated monocytes and platelets are probably poor competitors, since they possess few receptor copies despite their high affinity (see above). However, the concentration of free Stx locally elevated in blood supplying the STEC-colonized bowel sites during toxin translocation is bound to be reduced as the blood flows through other gut vessels, simply because of the huge amount of Gb3Cer receptors encountered on the enormous intestinal endothelial surface. Thus, blood refluxing from gut might contain PMN, carrying the toxins and low amounts of free Stx. In a short time the blood ejected by the heart completes its journey round the body together with passenger PMN. Therefore, when fresh toxins are present in the gut and continuously cross the epithelial intestinal barrier, PMN can progressively raise their receptor saturation until they or Stx disappear from the blood.

Another possible explanation might be that PMN encounter the toxins outside the circulation during the gut inflammation that follows the Stx-induced injury of the intestinal endothelial cells. The influx of neutrophils into the intestinal *lamina propria* and in the crypts of patients with HUS has been reported [77,151,152,153] and the inflammatory transmigration of PMN enhanced the translocation of Stx across intestinal epithelial cells [76]. Thus, PMN are found close to the basal surface of gut epithelial cells during the passage of toxins from the intestinal lumen. PMN carrying Stx could survive in the exudates, as they show a delay in the apoptotic program, being removed subsequently by lymphatic drainage eventually reaching the bloodstream. However, this last explanation seems unlikely since an abundant influx of PMN in the intestine, which can justify the mechanism of carriage of these leukocytes, was only observed in HUS cases in which the colon is severely involved.

## 8. Binding of Stx to PMN in Patients with HUS

After the first demonstration of the direct interaction Stx/PMN, Monnens’ group undertook a careful clinical study designed to demonstrate the role of such an interaction in HUS [120]. By means of indirect flow cytometry on PMN isolated from 11 patients with HUS, they found Stx on most of them. The binding was shown to be specific for PMN, as other circulating cells from those patients (lymphocytes, monocytes, erythrocytes) were negative with the exception of activated monocytes in a later phase of the disease [120]. They also observed that the percentage of positive PMN in patients decreased over time up to five days [120]. These clinical data were considered an important extension of the first observations *in vitro*, and a main contributor in comprehending the pathogenesis of HUS. Subsequently, the indirect flow cytometric method used for the detection of Stx bound to patients’ PMN [120] was adapted to white blood cells, isolated after erythrocytic lysis from whole blood, thus avoiding the complex procedure of PMN isolation [111]. PMN were easily recognized by gating them on morphology and by staining with monoclonal antibodies that react with granulocyte antigens CD16 and CD65. The new very rapid (few hours) and sensitive (femtomoles of toxin) version of the detection method also addressed the problem of false positive results, possibly due to direct interaction with PMN of mouse monoclonal antibodies specific for Stx. The addition to the assay of human serum to saturate Fc receptors avoided false positive Stx detection [111]. The drawback of this method is represented by the cross-reactivity of the mouse monoclonal antibodies to Stx1 (13C4) and Stx2 (BB12), which thus cannot discriminate the two toxin variants. These monoclonal antibodies, tested for the ability to neutralize toxin-induced cytotoxicity [59,127] or to inhibit the toxin-induced release of adenine from DNA (our unpublished observations), did not cross-react in reciprocal titrations of the two toxins. However, with respect to the latter assays in which toxins are free in solution, the situation is complicated by the presence of PMN during the cytofluorimetric assay. In the latter case, these antibodies must recognize the toxins present in binary (Stx1/receptor) or ternary (Stx2/HuSAP/receptor) complexes [110] and this might in part explain the cross-reactivity phenomenon. 

The kinetics of Stx in blood by this method and in feces by Vero cell cytotoxicity assay during the natural course of the disease was evaluated in a further study [121], showing a positive correlation between the amount of toxin detected in stools and the amount of Stx on PMN. In HUS patients a half-life of four days of Stx in blood was calculated and, surprisingly, toxins were still detectable in PMN for a median period of five days after they were no longer detectable in stools [121]. AlthoughStx-positive PMN show delayed apoptosis [112,119], they are not impaired in transmigration [114], hence in patients they must disappear from the blood within a few hours. However, *in vitro* co-incubation of fully-loaded PMN with empty PMN leads to the transfer of the toxic ligand between neutrophils [112]. Thus, it could be argued that in patients the Stx are transferred from old neutrophils to new mature cells entering the circulation from the bone marrow, in a sort of a relay race, extending the blood half-life of the toxins beyond the blood half life (6–7 h) or the life-span (1–2 days) of these cells. The implication of this long-lasting blood detection of Stx in the diagnosis of HUS and in defining the infective etiology is clear, since the windows of tests aimed at detecting Stx or STEC in feces are shorter. 

Since the cytofluorimetric assay used in the above-mentioned studies also provides a quantitative evaluation of the toxins bound to PMN, the relationships between the clinical course of HUS in patients and the amount of Stx detected in their blood samples have been sought [90]. In this study, patients with Stx-positive PMN (about 60%) were divided into two groups according to the saturation of Stx receptor in their PMN. Although no significant differences were observed in platelet and neutrophil counts, significant differences were observed in renal function: cases with high Stx levels on PMN (full receptor saturation) had slightly altered serum creatinine concentrations, whereas children with low Stx levels (about 1/3 receptor saturation) had values above the upper limit for age over several days during hospitalization. Moreover, patients with high Stx levels required less frequent dialysis with a shorter duration of dialytic treatment. These observations, albeit paradoxical, are consistent with those obtained in baboons challenged with intravenous infusion of high and low doses of Stx1 [154]. Although the animals belonging to both groups showed acute renal failure, the histopathological changes in the kidney were less pronounced in those animals challenged with high concentrations of toxin and which had no evidence of thrombotic microangiopathy in 60% of glomeruli. The low-dose group exhibited thrombotic microangiopathy in more than 90% of glomeruli accompanied by endothelial swelling (about 40%) and combined endothelial swelling and red corpuscle fragmentation (about 40%). The authors concluded that these findings reflected the picture observed in childhood post-diarrheal HUS [154]. Interestingly, in the same study, autopsies revealed edema of the brains of animals injected with the high-dose, whereas the brains of low-dose group exhibited a normal gross appearance and weight. Consistently, neurological complications were observed more frequently in HUS patients with high circulating levels of Stx on PMN [90]. The same behavior can be observed if the clinical data reported in the Monnens’ study on HUS patients described above [120] are stratified according to the amounts of toxins on PMN. In the Monnens’ study the saturation of toxin receptor on PMN was not reported, since the authors preferred to indicate the percentage of positive PMN over the whole cellular population. As shown in Table 3, by using this parameter and following the criteria indicated in [90] for the categorization of patients, the correlations between the two studies are clear-cut. 

**Table 3:** Clinical data ^a^ of patients with HUS and PMN positivity to Stx.

	High % Stx-positive PMN (mean ± SD, *n *= 4)	Low % Stx-positive PMN (mean ± SD, *n *= 3)	*t* Test
Creatinine (µM)	229 ± 124	627 ± 219	*p* < 0.05
Hb concentration (M)	5.0 ± 1.3	4.2 ± 1.8	*p* = 0.52
Platelets × 10^9^/L	36.3 ± 14.6	64.7 ± 28.2	*p* = 0.14

^a^ Stratification of the clinical data (first determinations) of patients in [120] following the criteria in [90]: cut-off ≤ 5%; low percentage of Stx-positive PMN >5% to 35%; high percentage of Stx-positive PMN > 35%).

No significant differences were observed in platelet counts and hemoglobin concentrations, whereas an inverse relationship between creatinine concentrations and percentage of positive PMN emerged: the lower the percentage of positive PMN, the higher the creatinine concentration (Table 3). Unfortunately, a patient with a low creatinine on presentation died during the study from neurological complications. This was indeed the patient with the high (96%) and most persistent percentage (92% after 5 days) of positive PMN, *i.e.*, a high-dose patient according to the findings of the more recent clinical study [90] and the data obtained with baboons [154]. However, by only analyzing the data in Table 3 one cannot draw conclusions about the severity of the HUS in those patients, since the original paper [120] did not report all the obvious complex clinical data recorded for each patient during their hospitalization. On the other hand, the main clinical characteristics reported in that study [120] support the idea [90] that the detection of the amounts of Stx on PMN might help clinicians identify patients at particular risk of neurological complication or with more relevant renal involvement.

Some years later, the same Dutch authors tried to explain the unreliability of their *in vitro* data on Stx binding to PMN, also casting some doubt on their clinical study, and in particular on the specificity of the cytofluorimetric assay for Stx detection on PMN [109]. They showed that 11 out of 16 patients without any symptoms of HUS, but needing hemodialysis, were positive to the cytofluorimetric assay for Stx detection, possibly for the known activation of PMN caused by this dialytic treatment [109]. Healthy control and non-HUS patients undergoing peritoneal dialysis were negative. Surprisingly, in the clinical study they had presented only patients undergoing peritoneal dialysis [120], a treatment which they considered safe for the detection of Stx on PMN [109], hence artefacts in Stx detection on PMN related to dialysis may also be excluded in their clinical study. It should be noted that another paper by the same authors aimed at detecting Stx on PMN of households of children with HUS showed that several persons living with patients had positive PMN even though they did not require any dialytic treatment [155]. This is consistent with the notion that only a low percentage of STEC-infected humans develop HUS and indicates a lack of correlation between dialytic treatment and detection of Stx bound to PMN. Consistently, a subsequent paper failed to confirm any correlation between positive cytofluorimetric assays and concomitant hemodialysis [90]. 

The inverse or direct relationship between the amount of toxins on PMN and renal or cerebral involvement is rather enigmatic. Various hypotheses can be advanced: (i) circulating PMN might capture free toxins preventing their binding to renal endothelium (sponge effect) but not to cerebral endothelium; (ii) the amounts of Stx on PMN membrane could reflect the differential avidity for toxins of renal endothelia but not of cerebral endothelia; (iii) PMN shuttle the gut-associated toxins to the kidney and brain but the endothelial responses to intoxication might be inversely related to the amounts of toxins delivered to renal endothelia and directly related in the case of cerebral endothelia. 

The last explanation is in agreement with previous observations *in vitro*. PMN loaded with different amounts of Stx, comparable with those observed in HUS patients, induced strikingly different responses in endothelial cells in terms of survival and production of pro-inflammatory cytokines [114]. Experimental transmigration of PMN carrying low amounts of Stx caused a relevant but not total inhibition of protein synthesis in endothelial cells and triggered a strong up-regulating effect on IL-8 and MCP-1 release without any effect on cell viability. Conversely, the transmigration of PMN with full saturation of Stx receptors almost completely blocked translation in endothelia with the concomitant impairment of IL-8 and MCP-1 induction and triggering of the apoptotic program (see also Figure 2). Therefore, patients with high Stx levels in PMN may develop sudden endothelial injuries that trigger apoptosis in intoxicated cells, without producing the slow self-amplifying pro-inflammatory cycle which might induce glomerular damage in the kidney. Notably, in the baboon model described above, the production of pro-inflammatory mediators was localized in the renal tissues [154] and high levels of urinary pro-inflammatory mediators in patients did not correlate with their blood levels [156,157]. 

The picture might be quite different when PMN-bearing toxins reach the blood-brain barrier which segregates blood and interstitial fluid. Brain capillary endothelial cells, unlike the fenestrated capillary in the kidney, are cemented by tight junctions [158] and interact intimately with astrocyte foot processes to form a barrier that selects and regulates transport of molecules in the central nervous system [159]. Sudden brain endothelial injuries imposed by large amount of toxins on PMN, rather than a slowly self-amplifying pro-inflammatory cycle as in renal endothelia, might well alter the delicate equilibrium of selective transport across the blood-brain barrier, eventually inducing the neurological complications observed in patients. Rabbit models of neuropathogenesis corroborate this hypothesis since intravenously injected Stx2 disrupted the blood-brain barrier [160]. The mechanism proposed is based on neuronal apoptosis caused by activated microglial cell [161]. Astrocytes activation may be secondary to endothelial damage and microvascular thrombosis induced by Stx in brain circulation [63].

## 9. The Nature of the Stx-Recognizing Receptors on PMN

Innate immunity, of which PMN are main actors, was regarded for a long time as a relatively non-specific system, with its main role being the killing of pathogens by phagocytosis and related mechanisms. In the past decades, the scenario has completely changed since several studies have revealed that the innate immune system possesses a high degree of specificity and is capable of discriminating between self and foreign pathogens at the molecular level. In fact, PMN, as well as macrophages, recognize molecular signatures common to different microorganisms but not to the host, which have been broadly defined as pathogen-associated molecular patterns (PAMPs). PMN recognize PAMPs by means of membrane or cytoplasmic receptors, called pattern-recognition receptors (PRR), such as Toll-like receptors (TLRs), C-type lectins receptors, NOD-like receptors and RIG-I-like receptors [162,163]. Some of these receptors are good candidates for membrane molecules capable of interacting with a class of different but analogous toxins, *i.e.*, Stx and related plant toxins. The interaction between PAMPs and a single PRR, although specific, is characterized by the recognition of a divergent collection of ligands, and the bound molecules are not necessarily internalized as is found for the Stx/PMN and the ricin A chain/PMN interactions. It is rather more an activation of a response leading to the production of cytokines required for the development of immunity and subsequent destruction of the pathogens. As stated above, PMN in HUS patients are activated and their functional state in the acute period of HUS is inversely correlated with the severity of renal damage: PMN from patients with the highest degree of renal failure showed the lowest level of markers of degranulation, intracellular myeloperoxidase and reactive-oxygen species production [104]. Clearly, identification of the nature of receptor would be the ultimate goal, and a recent study eliminated a number of receptor candidates since agonists of some TLRs (TLR1, TLR2, TLR5, and TLR6) or the mannose receptor are completely ineffective in displacing Stx from PMN in competition experiments [113]. Conversely, TLR4 improves the binding of Stx to primary human umbilical vein endothelial cells, indicating that it may also be exploited by bacterial toxins to interact with PMN [164], even though we lack evidence at the present time. TLR4 seems a good candidate since it is capable of interacting not only with LPS and the plant diterpene paclitaxel, but also with several proteins (fusion protein of respiratory syncytial virus, fibronectin and heat-shock proteins) [165]. A validation of this attractive hypothesis awaits the direct demonstration of the interaction Stx/TLR4 in human PMN.

## 10. Conclusions

The Stx/PMN interaction might resemble a double-edged sword since higher amounts of Stx on PMN might protect patients from acute renal failure, while contributing to neurological complications. It is not surprising, since neutrophils play a definite role in protecting hosts through their cooperation in innate and adaptive immunity. However, they also play a crucial role in the pathogenesis of numerous diseases [166]. Understanding the mechanisms of Stx delivery in blood, in particular the role of Stx bound to PMN or the role of free toxins, should help to develop evidence-based therapeutic strategies for preventing the onset of HUS or attenuate its clinical expression. Plasma exchange would be the elective therapy for clearing free plasmatic toxin, while the prevention of PMN/endothelium interactions with available drugs (selectin inhibitors, integrin inhibitors, chemoattractant receptor inhibitors) would be useful for PMN-bound Stx. However, a modification of the current standard of HUS management from purely supportive treatment to curative strategies requires clarification of the mode of delivery of the toxins in blood from studies on patients in the prodromal gastrointestinal phase, rather than on patients with overt HUS. In Italy, in 2010, a network connecting pediatricians, the Center for HUS control in Milan and the Regione Lombardia was set up for early enrolment of children with STEC-associated bloody diarrhea. This allows the study of the kinetics of Stx in the feces and blood of these patients by a daily sampling until recovery to focus on the crucial step of the transition between hemorrhagic colitis and HUS. It should be noted that in a recent Argentinean study this has been partially addressed [167] by screening about 2500 cases of diarrhea in humans for STEC infections. Almost 100 patients with STEC-associated gastroenteritis were enrolled and, during the observational study, 8 of them developed HUS. By means of a new validated ELISA the authors found free Stx2 in the sera of five patients who did not developed HUS and in three of those who developed HUS. Interestingly, the toxin appeared in blood just after the onset of diarrhea for a short time (48–72 h) and then disappeared before the development of HUS. This is consistent with the absence of detectable toxin in the sera of HUS patients reported in several studies [52,89,90]. However, in the Argentinean study no attempts were made to seek the presence of Stx on PMN. Moreover, five patients developing overt HUS were found negative for the detection of free Stx2 in sera during the prodromal phase, and no pathogenic mechanism was proposed. It is clear that this aspect of HUS pathogenesis is puzzling. What could be the crucial step in the transition between hemorrhagic colitis and HUS: the simple presence of Stx in blood, their amounts on PMN and/or their presence in plasma with subsequent rapid clearance, the presence of discrete peaks of toxins or a long-lived toxemia with lower toxin amounts? Only carefully conducted studies in patients in the prodromal phase of HUS or in well-suited animal models designed to identify, without any preconception, the pathogenetic process of HUS on the basis of our current knowledge, even though obtained in often conflicting reports, will be able to achieve this goal.
